# Small RNA Analysis in Sindbis Virus Infected Human HEK293 Cells

**DOI:** 10.1371/journal.pone.0084070

**Published:** 2013-12-31

**Authors:** Andras Donaszi-Ivanov, Irina Mohorianu, Tamas Dalmay, Penny P. Powell

**Affiliations:** 1 Norwich Medical School, University of East Anglia, Norwich, Norfolk, United Kingdom; 2 School of Biological Sciences, University of East Anglia, Norwich, Norfolk, United Kingdom; Virginia Tech, United States of America

## Abstract

**Introduction:**

In contrast to the defence mechanism of RNA interference (RNAi) in plants and invertebrates, its role in the innate response to virus infection of mammals is a matter of debate. Since RNAi has a well-established role in controlling infection of the alphavirus Sindbis virus (SINV) in insects, we have used this virus to investigate the role of RNAi in SINV infection of human cells.

**Results:**

SINV AR339 and TR339-GFP were adapted to grow in HEK293 cells. Deep sequencing of small RNAs (sRNAs) early in SINV infection (4 and 6 hpi) showed low abundance (0.8%) of viral sRNAs (vsRNAs), with no size, sequence or location specific patterns characteristic of Dicer products nor did they possess any discernible pattern to ascribe to a specific RNAi biogenesis pathway. This was supported by multiple variants for each sequence, and lack of hot spots along the viral genome sequence. The abundance of the best defined vsRNAs was below the limit of Northern blot detection. The adaptation of the virus to HEK293 cells showed little sequence changes compared to the reference; however, a SNP in E1 gene with a preference from G to C was found.

Deep sequencing results showed little variation of expression of cellular microRNAs (miRNAs) at 4 and 6 hpi compared to uninfected cells. Twelve miRNAs exhibiting some minor differential expression by sequencing, showed no difference in expression by Northern blot analysis.

**Conclusions:**

We show that, unlike SINV infection of invertebrates, generation of Dicer-dependent svRNAs and change in expression of cellular miRNAs were not detected as part of the Human response to SINV.

## Introduction

Small RNAs (sRNAs) are 20–30 nt non-coding RNAs that can regulate gene expression in a sequence-specific manner through a mechanism known as RNA interference (RNAi). Their biogenesis includes the processing of long double stranded RNA (dsRNA) into small RNAs (sRNAs) by Dicer. The sRNAs bind to homologous mRNAs and suppress gene expression. Importantly, in plants, insects and nematodes, RNAi can also serve as an innate immune response against viruses, as dsRNA produced by viruses as intermediates of replication is processed by a RNA Induced Silencing Complex (RISC) into small interfering RNAs (siRNAs) to target complementary viral mRNAs for destruction. It is still a matter of debate if RNAi plays a role in innate immunity in mammals [1], [2] because the sRNAs were only isolated at low concentration [3] and it is unclear whether the observed fragments are DICER dependent or not [4]. However, there is recent evidence that RNAi has a functional antiviral role in mouse embryonic stem cells and in tissues of suckling mice infected with Nodamura virus lacking an RNAi suppressor, although vsRNAs were reduced or absent in differentiated cells [5], [6]. Mammalian cells respond to virus infection through recognition of the dsRNA by two DEAD/box helicases, RIG-I and MDA-5. Activation of these helicases leads to induction of interferon and interferon stimulated genes (ISGs) which act in diverse ways to eliminate the virus [7]–[10] and it is thought that this interferon response has supplanted antiviral RNAi in higher organisms [1]. The components and function of the RNAi machinery are conserved in mammalian cells, although unlike plants and invertebrates, only one Dicer-like enzyme has been identified in human and its role in processing dsRNA replication-intermediates of mammalian viruses into vsRNAs is unclear [4], [11]. The development of high throughput sequencing technologies made possible the detection of low abundance sRNAs, facilitating the in depth study of vsRNAs. An initial study using deep sequencing from infection of a broad range of animal cells with six different RNA and DNA viruses showed the existence of some vsRNAs and changes in host miRNA expression [3].

SINV is an alphavirus in the Togovirus family which has a single stranded positive sense RNA genome of ∼12,000 nucleotides. The capsid protein enclosing the RNA is surrounded by a lipid bilayer with two transmembrane glycoproteins, E1 and E2. dsRNA is produced during replication, using the negative strand as a template for new copies of the genome. The first two-thirds of the genome contain the genes for the four nonstructural proteins, nsP1 to nsP4, which catalyze the replication and transcription of the viral RNAs [12]. The structural proteins, capsid and two envelope proteins are encoded in the latter one-third of the genome translated from a 26S subgenomic RNA. SINV is an arbovirus with a broad range specificity, infecting both insect and human cells and it is well established that in mosquitoes, RNAi is used as the primary antiviral defence. SINV-derived sRNAs have been detected and formed more than 10% of the sequenced sRNAs [13], [14]; recently, alphavirus-derived piRNA-like sRNAs have been found in mosquitoes [15], [16]. In addition, a novel class of endogenous siRNAs was discovered in Aedes aegypti mosquitoes infected by SINV. It is hypothesised that suppression of SINV replication by the mosquito RNAi is essential for the virus to survive in the mosquito vector [17]. However, little is known about RNAi in the regulation of SINV infection of mammalian cells.

In this study we looked for the presence of vsRNAs during a time course of SINV infection of human embryonic kidney 293 cells (HEK 293) and the changes in cellular miRNA profiles by high throughput sequencing. We found that the majority of reads derived from the human and not from the virus genome. In addition, based on the size class and complexity distributions of vsRNAs, we did not find any evidence that the reads could derive from Dicer processing. Furthermore, cellular miRNA expression profiles remained unchanged following SINV infection, and we conclude that modulation of the RNAi system is not an immediate early response to SINV infection of human cells.

## Materials and Methods

### Cells and Viruses

All cells were maintained at 37°C in 5% CO_2_. HEK 293 cells derived from human embryonic kidney isolate were grown in DMEM-Glutamax (Invitrogen, Dulbecco/Vogt modified Eagle's medium) with 5% non-essential amino acids, 10% FCS and penicillin-streptomycin. Baby Hamster Kidney (BHK) cells were maintained in DMEM/F-12 (Invitrogen) with 5% non-essential amino acids, 10% FCS and penicillin-streptomycin.

Sindbis Virus (SINV) AR339 was received from John Fazakerly, The Pirbright Laboratory, UK. TR339 infectious cDNA clone was from William Klimstra, University of Pittsburgh, Pittsburgh, PA. Rabbit anti-Sindbis virus polyclonal antibody was a gift from Sondra Schlessinger, Washington University Medical School St Louis, MO.

### Sindbis virus (SINV) production

SINV A339V stocks were grown in BHK cells and then virus was adapted to grow in HEK 293 cells by up to three serial passages to make high titre HEK 293 stocks. Virus growth was slower in HEK 293 cells compared to BHK cells, with a replication time of 8hpi at each passage, and viral titres increased. Only low passage number virus was used to avoid overproduction of defective interfering particles and to limit sequence changes in the adapted virus. The final viral sequence used for inoculation was assembled using reads from the Illumina sequencing. Virus protein E2 was detected by Western blot and immuno-staining using an anti SINV antibody, and by using RT-PCR with SINV specific primers. AR339 SINV was concentrated by polyethylene glycol precipitation. (PEG-IT; System Biosciences) PEG-IT was added to the media in a 1:5 dilution and incubated at 4°C overnight. The precipitate was collected by centrifugation with 3000 g for 30 minutes at 4°C, and resuspended in OPTIMEM (Invitrogen).

### RNA isolation

Trizol (Invitrogen) was used to extract SINV RNA, both for total RNA isolation and sRNA. For isolation of sRNA the samples were incubated at −20°C in isopropanol overnight. The mirVana (Ambion) kit was used according to the manufacturer's instructions to isolate total RNA for Illumina Solexa sequencing of sRNA.

### High Throughput Illumina Solexa sequencing

High-throughput sequencing was carried out as previously described [18] using v1.5 of the Illumina adapters. The total RNA was isolated, reverse transcribed with v1.5 adapters and amplified (18 cycles). The 90–110 nt (75 nt adapter-adapter) band was excised, corresponding to 15 to 35 nt RNA sequences and the sRNA library was sent to Baseclear (www.baseclear.nl) for sequencing on the Illumina/Solexa GA II [19],[20].

### Northern blotting

Total RNA was isolated from samples using Trizol (Invitrogen). Electrophoresis, transfer and blotting were performed as previously described [21]. After chemical crosslinking, the membranes were hybridised overnight in ULTRAhyb-Oligo hybridisation buffer (Ambion) containing either γ-P^32^ ATP labelled primer probes, or γ-P^32^ ATP locked nucleic acid (LNA) probes (incubated at 37°C and 62°C respectively), or with α P^32^-CTP labelled riboprobes (incubated at 64°C), and imaged using Fuji phosphorimaging screen. The sequences used are

SINV genomic and subgenomic (position 7568–7631): GTATTAGTCAGATGAAATGTACTATGCTGACTATTTAGGACCACCGTAGAGATGCTTTATTTCC, LNA probe sequence (position 6614-6633): GGATAGATTCGTCATGGACA. U6 (TCATCCTTGCGCAGGGGCCA) was used as loading control.

The miRNAs selected for validation and the sequences used as probes are as follows:

mir 29a:TAACCGATTTCAGATGGTGCTA; mir 34: ACAACCAGCTAAGACACTGCCA;

mir 10:ACAAATTCGGTTCTACAGGGTA;

mir 19:TCAGTTTTGCATGGATTTGCACA; let 7:AACTATACAATCTACTACCTCA;

mir 92:GGAGGCCGGGACGAGTGCAATA; mir 378: GCCTTCTGACTCCAAGTCCAGT; mir196: CCCAACAACATGAAACTACCTA;

mir 197: GCTGGGTGGAGAAGGTGGTGAA.

### Bioinformatics methods

For the analysis we used the GenBank reference sequence for the Sindbis virus gi|9790313|ref|NC_001547.1 [22] and the latest available sequence for the Human genome (hg19) [23].

The preliminary analysis of the sequenced libraries was conducted using the UEA sRNA Workbench [24]. The adaptor removal, quality check and genome matching were conducted as described in [25]. The accepted reads (the entire length of the reads) were mapped with no mis-matches against the human genome, or with up to 2 mis-matches against the SINV genome. The normalization was conducted using the proportional scaling approach, “reads per million” (RPM) [26]. The identification of miRNAs was conducted using miRCat [27], with standard parameters for human samples and miRprof using all mature and precursor sequences of miRNAs deposited in miRBase [28] as input. The statistical analysis of the properties of sRNAs was conducted in R using the standard *stats* package.

## Results

### Sindbis virus is efficiently replicated in human HEK 293 cells

The role of RNAi in the defence of mammalian cells against RNA viruses is not yet established. SINV is a valuable research model of a positive stranded RNA virus, which cycles through arthropod and non-human vertebrate hosts. SINV AR339 is an attenuated laboratory strain, first isolated from mosquitoes, which has been adapted to replicate in BHK cells, and which does not cause disease in humans [29].

Virus generated in BHK cells was adapted through two passages through HEK 293 cells. Virus showed a slower kinetics of replication and secretion compared to BHK cells with virus secretion beginning by 8hpi, and an increased viral titre was obtained with each passage. The SINV genome has been shown to have lower rates of evolution than other RNA viruses [30] and adaptation to various cells has been shown to result from mutations in E2 to enhance binding and infection [31].

**Figure 1:**
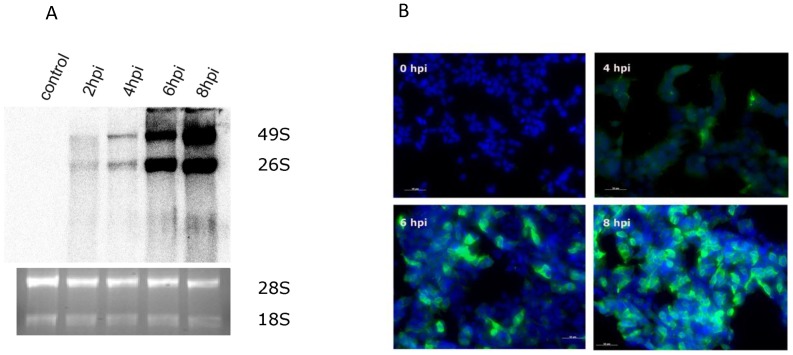
Infection and replication of SINV in HEK293 cells. (A). Northern blot showing SINV full length genome (49 S) and subgenomic (26 S) positive strand RNA rapidly accumulates in HEK293 cells from 2hpi and increasing through 8 hpi. The probe consisted of P32 end-labelled primers at positions 7568–7631 in the genome. The 28 S and 18 S RNA bands stained with ethidium bromide are displayed to demonstrate equal loading. (B) Immunostaining of SINV in HEK293 cells using rabbit anti E2 glycoprotein antibody detected SINV E2 translation increasing over 4, 6 and 8 hpi with over 95% of cells infected with well-defined replication centres (visualised with an anti-rabbit secondary antibody labelled with Alexa488 and nuclei are shown with DAPI).

RNA was isolated at different time points (2, 4, 6, 8hpi) in HEK293 cells and virus replication was detected by Northern blotting with SINV specific probes. The presence of full length transcripts at 49S and the sub genomic transcript at 26 S demonstrated efficient replication starting between 2 and 4hpi and increasing through 8hpi (Figure 1A). During the same time course the envelope glycoprotein E2 was observed by immunofluorescence at 4hpi and increasing through 8hpi (Figure 1B) demonstrating that SINV effectively and rapidly infected and replicated in HEK293 cells. Based on this information the time points for sRNA sequencing were chosen at 4hpi and 6hpi at times when the highest level of dsRNA replication intermediates as substrates for Dicer can be observed. After 8hpi virus is secreted into the media (increased intracellular virus production, figure 1B) and the apoptotic process initiates after approximately 24hpi (data not shown). Therefore an earlier time point, 6hpi, was chosen to avoid any chance of RNA degradation due to initiation of apoptosis.

### High-throughput sequencing shows viral sRNAs in very low abundance in SINV infected HEK 293 cells

**Figure 2:**
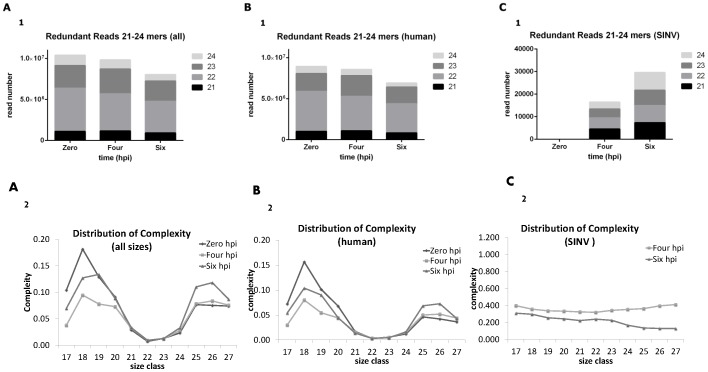
Size class and complexity distributions of sequencing reads. Size distribution and complexity are shown for (A) all reads, (B) reads mapping to the Human genome, (C) reads mapping to the SINV genome. Subplots A1, B1 and C1 show the read number for 21–24 mers at 0, 4 and 6hpi. Subplots A2, B2 and C2 show the complexity for each size class from 17–27 mers, where the complexity is the ratio of non-redundant reads to redundant reads.

RNA was isolated from SINV inoculated cells at 0, 4 and 6hpi. cDNA libraries were generated for the sRNA content of the cells and sequenced using Illumina GA II, which yielded between 29.1million (M) and 30.5 M reads per sample. The size class and complexity (defined as the ratio of non-redundant to redundant reads [25]) distributions for all reads for which the adapter sequence was identified is presented in figure 2A. We observed a preference for sequences of lengths 22–23 nt, which are also characterised by a low complexity indicating a low number of unique reads with high abundance.

Next, the sequences were mapped to the human and SINV genomes, respectively, using PatMaN [32] revealing that more than 83% of sequences mapped to the human genome (HUM reads), and 0.8% sequences mapped to the SINV genome (SINV reads). The size class and complexity distributions for all reads are shown in Figure 2A. The size class and complexity distributions of the HUM reads (figure 2B) are similar to the overall distributions, preserving the properties of the 22–23 nt reads. In addition, a low complexity for the HUM 22mers indicates miRNAs – subplot B2 (subsequent analysis revealed that more than 60% of these reads correspond to known miRNAs). The SINV reads (figure 2C) showed an even distribution for all size classes, suggesting that these reads are not Dicer-derived. This hypothesis is also supported by the uniformity of the complexity index (i.e. the complexity was similar for all size classes in the 4hpi and 6hpi samples). The size class distribution and complexity analyses were also conducted separately on each strand and the conclusions were the same (figure S1). No conclusion was based on the complexity distribution for the mock sample because of the extremely low number of SINV reads (20 reads) present. In addition the ratios of vsRNAs mapping to positive and negative strand were 4:1 at 4hpi and 20:1 at 6hpi, similar to what would be expected at these time points for the ratio of positive and negative RNAs [33], [34].

**Figure 3:**
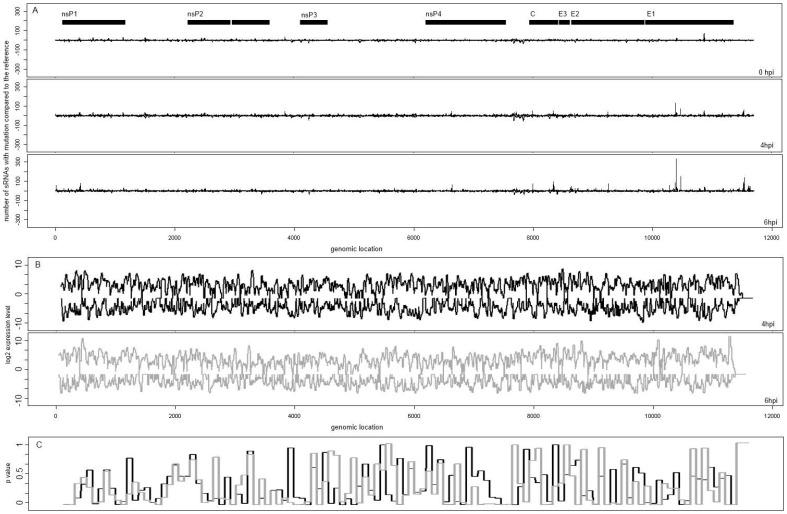
Analysis of SINV matching reads indicate degradation. (A) Nucleotide variation across the genome (x axis) at 0, 4 and 6 hpi on both positive and negative strands is indicated by the number of unique sRNAs (0 to 300 and 0 to −300, respectively) which vary at a given position (y axis). This analysis indicates a high similarity of the Sindbis variants present in the cell to the reference sequence. The open reading frames are indicated by black boxes at the top. (B) Variation of expression level (log2 scale) for SINV matching reads. The figure shows the distribution of viral reads along the SINV genome (x axis). Positive values on y axis indicate the abundance of reads mapping to the positive strand of the virus, negative values indicate the abundance of reads mapping to the negative strand. Black represents the 4hpi reads, grey represents the 6hpi reads. (C) Variation of p Value presented on the y axis for a *x* ^2^ significance test on the size class distribution compared to a random uniform distribution for windows of length 100 nt along the SINV genome (x axis). Black represents 4hpi and grey represents 6hpi sRNA samples.

To investigate whether the passage resulted in changes to the reference sequence of the viral inoculum, meaning that some vsRNAs may be missed, even if 2 mismatches were allowed, we assembled the vsRNAs at each time point into contigs which were then compared to the reference sequence (figure 3A). The sequence of the adapted virus was similar to the NCBI reference sequence; in 4hpi and 6hpi one significant nucleotide polymorphism (SNP) was detected in E1 at position 10393. This SNP was a change from G to C or A (figure S2) which led to a valine to leucine or isoleucine substitution. This is a conservative change which would not be likely to affect protein function. In addition there were no changes in sRNA size class distribution or complexity between the adapted and reference genomes.

**Figure 4:**
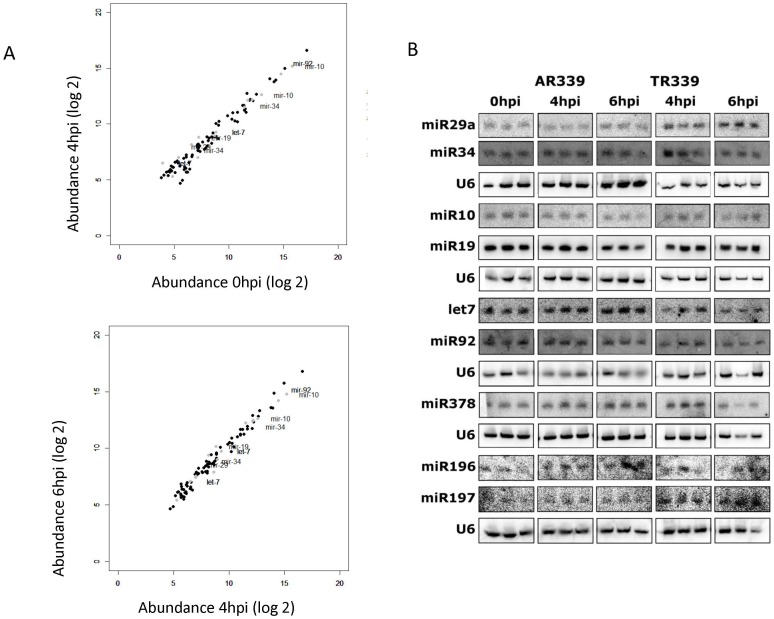
Human miRNA expression profiles remain unchanged during early SINV infection (0, 4 and 6phi). (A) Scatter plots of miRNA expression levels indicate no significant change in expression in HEK293 cells between 0 and 4 hpi (top panel) and 4 and 6 hpi (bottom panel). x and y axes show the normalised expression levels of the miRNAs (log2 scale). (B) Northern blot validation of candidate miRNAs in SINV AR339 and infectious clone TR339 indicate no change in expression. The Northern blots are biological triplicates. The equal loading is shown by U6.

To investigate the hypothesis, that the SINV reads are not Dicer-derived reads, we analysed the distribution of expression (sum of abundances of SINV reads for all positions) for the whole genome (figure 3B) and conducted a *x* ^2^ analysis applied on the size class distribution compared to a random uniform distribution, for windows of length 100 nt (figure 3C). The purpose of identifying regions which show a preference for a size class is that these regions are likely to be excised in a precise manner through the RNAi pathway [35]. This analysis revealed highly significant regions (i.e. regions for which the size class distribution was significantly different from a random uniform distribution, the p Value was below 0.05 in both 4hpi and 6hpi samples), and regions for which the size class distribution was very similar to a random uniform distribution (p Value above 0.7, in both 4hpi and 6hpi samples). We attempted the validation of reads coming from the highly significant regions, but their abundance was found to be below the detection limit of Northern blot analysis using primers or LNA probes (data not shown). The detection limit for the miRNA northern blots (shown in figure 4B) was approximated to 100 normalized reads per million; the maximum abundance of specific fragments matching to SINV is below 70. Increasing the number of mis-matches between the reads and the reference genome (0, 1, 2, 3 mis-matches) did not change the conclusions.

To better understand the interaction between the virus and the host, we computed the number of reads that could match to both the virus genome and the human genome. 5% of the SINV reads matched to both genomes. All of the reads were low abundance and many variants (sequences with high similarity and less than 4 mis-matches to the considered read) were present on the SINV alignment, suggesting that the reads have a higher probability to be produced by the virus RNA, rather than the human genome. Figure 3B shows the lack of hotspots, which suggest the absence of specific cleavage of the viral genome and the lack of location specificity on the SINV genome. In addition, the uniform distribution of each size class for reads on both strands supports the hypothesis that the reads are not Dicer derived fragments.

### SINV infection does not modulate the cellular miRNA expression

Since the majority of reads mapped to the human genome (>8 M and >6 M reads in the first two samples and in the third, respectively), we also investigated the changes induced by the virus infection in the cellular sRNAome. First, we identified miRNAs using miRCat [27] and mirProf [24]; out of the 110 predicted miRNAs, 92 (including variants) were conserved and 18 were novel. To further investigate the sRNAome changes, other properties of miRNA loci were analysed. First, using all human miRNAs from miRBase [28] for which we could identify at least 3 reads in the samples, the distribution of signal across the precursors was analysed. For all miRNAs more than 90% of the signal was consistently concentrated on the miRNA and miRNA*, in all three samples. Next the size class distribution on the precursors was analysed using a *x* ^2^ test (the approach was similar to the one used for the analysis of the whole SINV genome). The approach was applicable since the distribution of pre-miRNA lengths shows little variation around the 100 nt mark, which was used for the SINV genome. Under the assumption of random uniform distribution, all of the pre-miRNAs had significantly different size class distributions, biased on 22mers. The distributions for samples 4 hpi and 6 hpi were statistically the same as for the 0 hpi sample, suggesting no influence of the virus infection to modulate the miRNA expression (figure 4A). The scatterplot on annotated miRNAs shows that they display little variation between the 0 hpi, 4hpi and 6 hpi time points. Although the variation was small, we selected twelve miRNAs, showing the most difference in expression by deep sequencing (Figure 4A), as candidates for Northern blot analysis; in addition, these miRNAs have been linked to antiviral immunity [36]–[47].

Northern blot validation of the sequencing results (Figure 4B) was carried out on HEK293 cells which were infected with SINV AR339 and SINV TR339 (an infectious clone of the same virus) in biological triplicates at moi of 8; total RNA was isolated at 0, 4, and 6 hpi. Three microRNAs were under detection limit and nine showed no changes in expression levels confirming the conclusions from sequencing.

These results show that the Sindbis virus infection does not change miRNA expression in HEK293 cells at a time when miRNAs may regulate an innate immune response or affect viral replication [13].

## Discussion

This study investigated the role of RNAi during SINV infection in mammalian cells, in the light of the well-established role of RNAi in SINV infection of insect vectors [13], [14], [46].

To date several modes of action of RNAi as a response to viral infection have been proposed [2]: (1) RNAi produces svRNAs that can target the virus genome (or transcripts in the case of DNA viruses), (2) the viral genome encodes miRNA-like regions that are processed by RNAi into miRNAs which can in turn target the genes of the host, (3) the host genome encodes miRNAs which can modulate viral replication through RNAi and (4) viral infection can influence the expression of cellular miRNAs. The effect of RNAi can therefore be antiviral or beneficial to the virus.

An example of the antiviral effect of the RNAi machinery, which illustrates the first mode of action, is the systemic RNAi response against SINV in Drosophila [46]. The second mode of action is illustrated by the herpes virus family and other large DNA viruses which encode miRNAs that target cellular innate and acquired immunity factors [36]. For example, miRNAs encoded by EBV target the pro-apototic factor PUMA, miRNAs encoded by KSHV down regulate MyD88; and miRNAs encoded by HCMV inhibit RANTES expression [36], [37], [44]. The third mode of action is illustrated by Hepatitis C Virus, for which the liver-specific miR-122 has been shown to have a stimulating effect on viral replication [42]. EBV infection is an example of the fourth mode of action (viral infection modulates cellular miRNAs expression). EBV strongly induces miR155 in B cells to promote cell transformation [39].

In this context, we studied sRNAs during SINV infection of mammalian cells to understand in which of these categories SINV can be classified. Sequencing showed that there were no svRNAs in the 20-25 nt range which could indicate the processing of the dsRNA virus replication intermediate by the RNAi machinery. This would suggest that there is no self targeting of viral RNA. This is in contrast to SINV infection in mosquito cells where SINV is a substrate for RNAi [13], [38] and svRNAs prevented viral spread between cells in Drosophila [46]. In mosquitoes variations in levels of Ago2, DICER2 and other components of RISC were observed during virus infection, indicating that the virus can modulate the RNAi system [13]. In this study we cannot rule out if there are changes in AGO2, DICER and other RISC components at protein level.

In addition, the distribution of 20–25 nt fragments mapping to the viral genome indicated that there are no viral miRNAs encoded in the viral genome that could affect gene expression of either host or virus. Viral miRNAs have been well documented in DNA viruses, such as herpes viruses and adenoviruses [40], but viral miRNAs have not been described for RNA viruses except for the retrovirus bovine leukemia virus (BLV) [45].

In addition, our sequencing results showed that host miRNAs were not differentially expressed in SINV infected cells and this was confirmed by Northern blot. We chose as candidates those miRNAs that have been linked to anti-viral immunity. MiRNA 29a has already been linked to several defences against pathogens. It is shown to regulate the immune response to intracellular bacterial infection through IFNγ modulation [48].The level of miR29 increases 50-fold in A549 cells in response to influenza infection, which leads to IFNλ and COX2 up-regulation [49]. In addition, miR-29 down regulates the expression of Nef protein of HIV-1, and it interferes with HIV-1 replication [50]. miR378 was also shown to be targeting HIV-1 genes [41]. mir34 was shown to be an important modulator of innate immune response through the regulation of IFNβ expression [47]. Deep sequencing data of Pseudorabies virus showed that the large latency transcript (LLT) functions as a primary microRNA precursor (pri-miRNA) encoding 11 distinct miRNAs in the PRV genome [51]. KSHV encodes 17 miRs, derived from 12 pre-miRs, with one regulating the NF-κB pathway by reducing the expression of IκBα protein, an inhibitor of the NF-κB complex [52]. miR19 and let7 are important regulators of inflammatory responses as they upregulate NF-κB activity[53]. Let7 also modulates the innate immunity through the regulation of IFNβ expression [48]. The miR17/92 cluster has been shown to regulate Epstein-Barr virus gene expression [54]. miR196 can effectively repress hepatitis C virus gene expression and replication [55]. miR197 targets the tumour suppressor protein FUS1 [56]. Moreover, this mode of action has been associated with some RNA viruses, for example the picornavirus enterovirus 71 (EV71) activates transcription of miR-141, which in turn suppresses translation of the cap-binding protein eIF4E in order to inhibit cap dependent translation [43].

Although we found that SINV infection did not up-regulate host miRNAs, it does not preclude pre-existing cellular miRNAs influencing SINV replication, as in the case of the RNA virus HCV where the liver specific miR-122 stimulates viral translation [42]. It is possible that SINV-induced changes in endogenous miRNAs occur only in its arthropod host species, such as mosquitoes [15], [17] and its nonhuman vertebrate host.

## Conclusions

Our work adds to the understanding of interaction between the alphavirus SINV and its human host through RNAi. We conclude that the svRNAs detected by deep sequencing were not generated by Dicer. The svRNAs may be random degradation products or the viral RNA may be specifically targeted by the cell but at present cannot be ascribed to a specific degradation pathway. Although we know that interferon is not induced by SINV, at least at early time points [57], RNase L could be a candidate via 2′, 5′- oligodenylate synthetase OAS to generate these degradation products [58]. Host miRNA profiles remained unchanged during the early stages of the infection and we conclude that they do not contribute to the response against SINV infection. In light of recent studies [5], [6] one possibility is that SINV encodes a potent inhibitor of RNAi.

## Supporting Information

Figure S1 **Size class and complexity distributions of reads matching to SINV genome with up to 2 mis-matches (2 mm)**, positive strand (A) and negative strand (B) shown separately for 0, 4 and 6 hpi. There is no preference for a size class in the redundant and non-redundant distributions and the complexity (varying between 0 and 1) remains unchanged.
(TIFF)

Figure S2 **Single nucleotide polymorphism (SNP) observed in E1 during viral replication.** The number of unique sRNAs with mutation compared to the SINV reference are presented on the Y axis for all four nucleotides. The SNP changes G to C or A, but never a T and the corresponding amino-acid changes from valine (GTC) to leucine (CTC) or isoleucine (ATC) in E1.
(TIF)
