# Supplementary figures and images for: Small RNA Analysis in Sindbis Virus Infected Human HEK293 Cells
Source: PLoS One. 2013 Dec 31;8(12):e84070. doi: 10.1371/journal.pone.0084070 (PMC3877139; doi:10.1371/journal.pone.0084070)

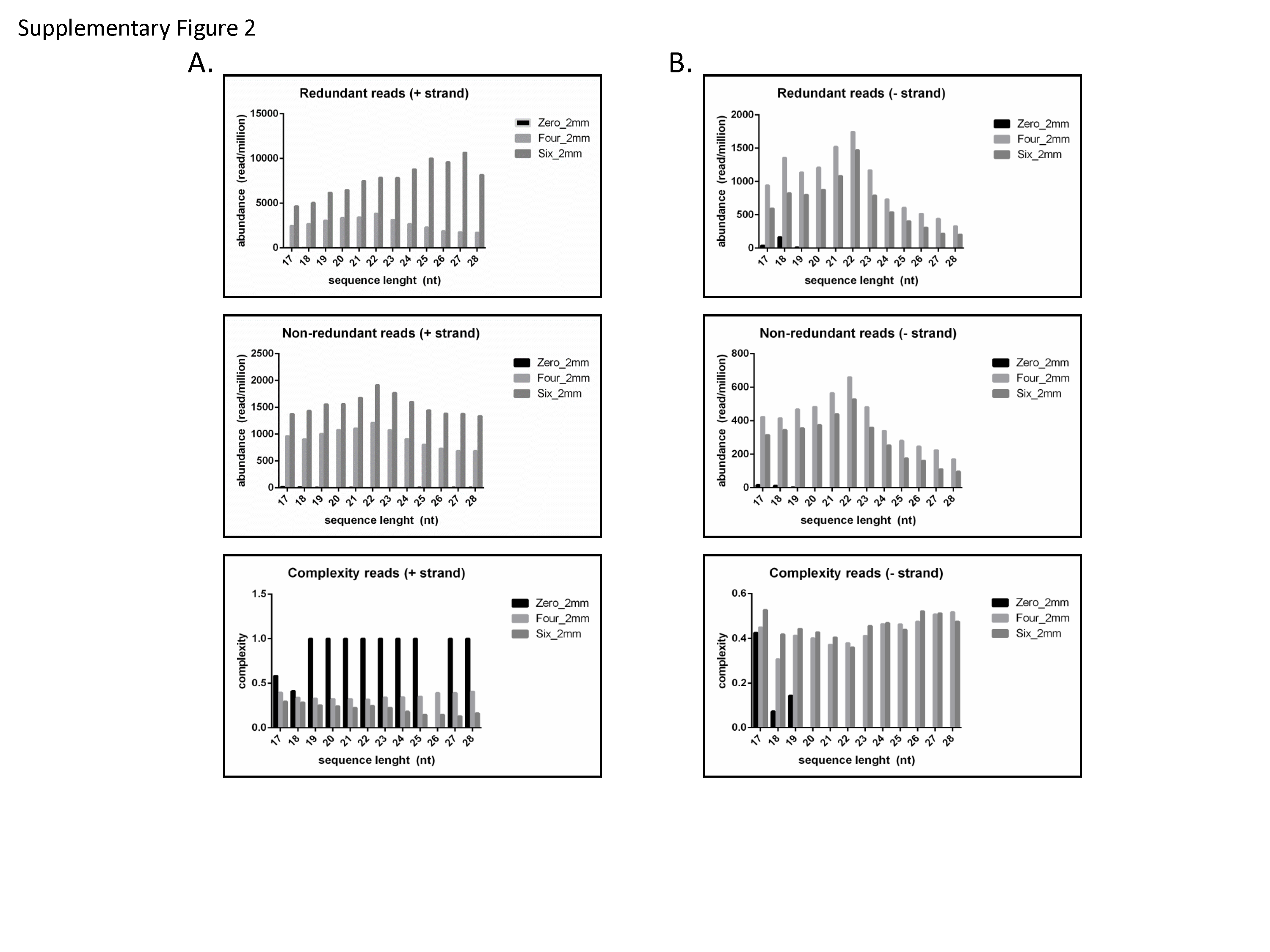

Supplement: Figure S1 — Size class and complexity distributions of reads matching to SINV genome with up to 2 mis-matches (2 mm), positive strand (A) and negative strand (B) shown separately for 0, 4 and 6 hpi. There is no preference for a size class in the redundant and non-redundant distributions and the complexity (varying between 0 and 1) remains unchanged. (TIFF) [file pone.0084070.s001.tiff]

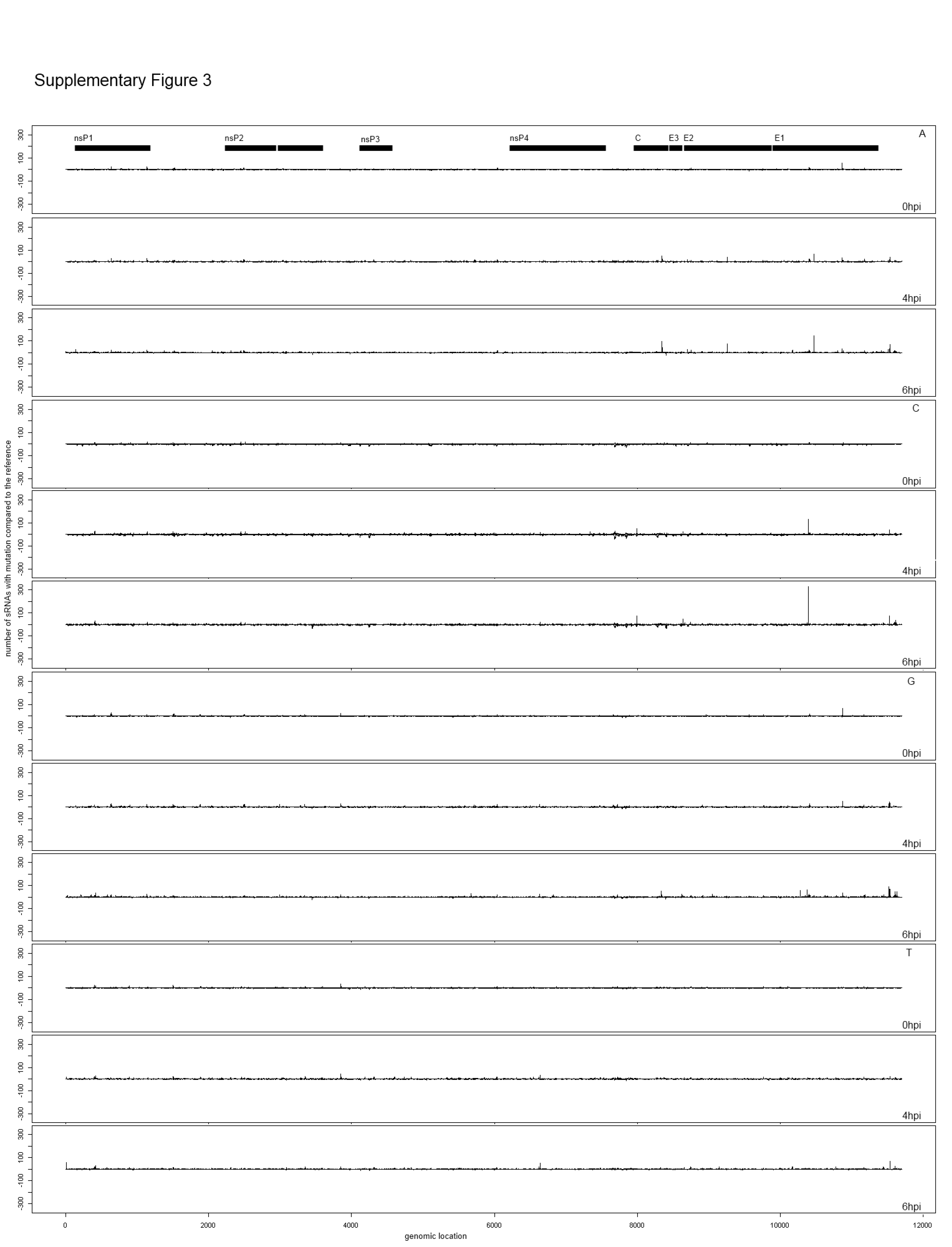

Supplement: Figure S2 — Single nucleotide polymorphism (SNP) observed in E1 during viral replication. The number of unique sRNAs with mutation compared to the SINV reference are presented on the Y axis for all four nucleotides. The SNP changes G to C or A, but never a T and the corresponding amino-acid changes from valine (GTC) to leucine (CTC) or isoleucine (ATC) in E1. (TIF) [file pone.0084070.s002.tif]
